# Supplementary material for: Modeling glioblastoma heterogeneity as a dynamic network of cell states
Source: Mol Syst Biol. 2021 Sep 16;17(9):e10105. doi: 10.15252/msb.202010105 (PMC8444284; doi:10.15252/msb.202010105)
Supplement: Supplementary file 5 — Source Data for Figure 3 [file MSB-17-e10105-s001.zip › Figure3A_sourcedata/GSEA_3065/nosigdb_state2.GseaPreranked.1622718419215/RICHARDS_NEFTEL_CELL_2019_G2_M.html]

Details for gene set RICHARDS\_NEFTEL\_CELL\_2019\_G2\_M[GSEA]

|  || Dataset | state2 |
| Phenotype | NoPhenotypeAvailable |
| Upregulated in class | na\_pos |
| GeneSet | RICHARDS\_NEFTEL\_CELL\_2019\_G2\_M |
| Enrichment Score (ES) | 0.87887377 |
| Normalized Enrichment Score (NES) | 2.5822523 |
| Nominal p-value | 0.0 |
| FDR q-value | 0.0 |
| FWER p-Value | 0.0 |
Table: GSEA Results Summary

  

Fig 1: Enrichment plot: RICHARDS\_NEFTEL\_CELL\_2019\_G2\_M      
 Profile of the Running ES Score & Positions of GeneSet Members on the Rank Ordered List

  

| PROBE | GENE SYMBOL | GENE\_TITLE | RANK IN GENE LIST | RANK METRIC SCORE | RUNNING ES | CORE ENRICHMENT || 1 | H2AFZ |  |  | 8 | 0.735 | 0.0622 | Yes |
| 2 | CENPF |  |  | 34 | 0.518 | 0.1039 | Yes |
| 3 | SMC4 |  |  | 42 | 0.489 | 0.1451 | Yes |
| 4 | TMPO |  |  | 43 | 0.489 | 0.1870 | Yes |
| 5 | KPNA2 |  |  | 50 | 0.472 | 0.2269 | Yes |
| 6 | CCNB1 |  |  | 68 | 0.420 | 0.2611 | Yes |
| 7 | KIF4A |  |  | 84 | 0.393 | 0.2932 | Yes |
| 8 | TUBB4B |  |  | 125 | 0.340 | 0.3180 | Yes |
| 9 | KIF11 |  |  | 129 | 0.335 | 0.3464 | Yes |
| 10 | TACC3 |  |  | 130 | 0.334 | 0.3751 | Yes |
| 11 | CCNA2 |  |  | 135 | 0.331 | 0.4031 | Yes |
| 12 | ANP32E |  |  | 136 | 0.330 | 0.4314 | Yes |
| 13 | ARHGAP11A |  |  | 145 | 0.321 | 0.4581 | Yes |
| 14 | CKAP2 |  |  | 151 | 0.308 | 0.4840 | Yes |
| 15 | CDCA3 |  |  | 160 | 0.304 | 0.5093 | Yes |
| 16 | ECT2 |  |  | 171 | 0.297 | 0.5337 | Yes |
| 17 | MZT1 |  |  | 174 | 0.295 | 0.5588 | Yes |
| 18 | CCNB2 |  |  | 190 | 0.286 | 0.5818 | Yes |
| 19 | CDC20 |  |  | 193 | 0.284 | 0.6059 | Yes |
| 20 | HMGB3 |  |  | 200 | 0.282 | 0.6295 | Yes |
| 21 | FOXM1 |  |  | 202 | 0.282 | 0.6536 | Yes |
| 22 | KIF2C |  |  | 252 | 0.251 | 0.6699 | Yes |
| 23 | CKAP2L |  |  | 327 | 0.224 | 0.6812 | Yes |
| 24 | MXD3 |  |  | 328 | 0.224 | 0.7004 | Yes |
| 25 | RACGAP1 |  |  | 333 | 0.222 | 0.7191 | Yes |
| 26 | BUB1 |  |  | 335 | 0.222 | 0.7380 | Yes |
| 27 | PLK1 |  |  | 364 | 0.213 | 0.7533 | Yes |
| 28 | AURKA |  |  | 375 | 0.210 | 0.7703 | Yes |
| 29 | NCAPD2 |  |  | 434 | 0.193 | 0.7806 | Yes |
| 30 | TROAP |  |  | 435 | 0.193 | 0.7972 | Yes |
| 31 | SPAG5 |  |  | 439 | 0.192 | 0.8133 | Yes |
| 32 | KNSTRN |  |  | 483 | 0.183 | 0.8244 | Yes |
| 33 | CDC25B |  |  | 519 | 0.178 | 0.8360 | Yes |
| 34 | BUB1B |  |  | 572 | 0.168 | 0.8448 | Yes |
| 35 | KIFC1 |  |  | 577 | 0.167 | 0.8587 | Yes |
| 36 | CDCA8 |  |  | 634 | 0.158 | 0.8663 | Yes |
| 37 | TUBA1C |  |  | 644 | 0.157 | 0.8789 | Yes |
| 38 | TTK |  |  | 831 | 0.135 | 0.8706 | No |
| 39 | PSRC1 |  |  | 1023 | 0.118 | 0.8604 | No |
| 40 | KIF20A |  |  | 1094 | 0.112 | 0.8626 | No |
| 41 | RANGAP1 |  |  | 1157 | 0.107 | 0.8652 | No |
| 42 | REEP4 |  |  | 1734 | 0.076 | 0.8103 | No |
| 43 | OIP5 |  |  | 2175 | 0.058 | 0.7685 | No |
| 44 | ARL6IP1 |  |  | 2421 | 0.050 | 0.7467 | No |
| 45 | GPSM2 |  |  | 5127 | -0.002 | 0.4590 | No |
Table: GSEA details [plain text format]

  

Fig 2: RICHARDS\_NEFTEL\_CELL\_2019\_G2\_M: Random ES distribution      
 Gene set null distribution of ES for **RICHARDS\_NEFTEL\_CELL\_2019\_G2\_M**
